# Clinicopathological characteristics of invasive stratified mucinous carcinoma of the cervix and the expression and clinical significance of SLC7A11, SLC3A2 and PD-L1

**DOI:** 10.3389/fonc.2024.1492498

**Published:** 2024-12-30

**Authors:** Changyu Lu, Wei Zhu, Xiahui Han, Xiuzhen Du, Hui Zhang, Qin Yao, Ting Liu, Ce Zhang

**Affiliations:** ^1^ The Affiliated Hospital of Qingdao University, Qingdao, China; ^2^ Qilu Hospital, Shandong University, Jinan, China; ^3^ The Second Affiliated Hospital of Shandong First Medical University, Tai’an, China; ^4^ Department of Obstetrics and Gynecology, Qilu Hospital, Shandong University, Jinan, China; ^5^ Shandong Second Medical University, Weifang, China

**Keywords:** invasive stratified mucus of the cervix produces carcinoma, PD-L1, SLC7A11, SLC3A2, immune checkpoint inhibitors, ferroptosis

## Abstract

**Introduction:**

Invasive Stratified Mucin-producing Carcinoma (ISMC) of the cervix is a newly named cervical adenocarcinoma associated with Human Papilloma virus (HPV). Due to its relative rarity, clinical data, pathological features, and molecular characteristics of ISMC are still under exploration. This study aims to retrospectively analyze the clinical data and pathological features of ISMC patients, summarizing the clinical and pathological morphological characteristics of ISMC. Immunohistochemistry for SLC7A11, SLC3A2, and PD-L1 will be performed on tumor tissues from ISMC patients to preliminarily explore potential therapeutic targets for ISMC.

**Methods:**

We retrospectively reviewed the electronic medical records and pathological slides of 22 ISMC patients, and performed immunohistochemical staining for solute carrier family 7 member 11 (SLC7A11), solute carrier family 3 member 2 (SLC3A2), and programmed death-ligand 1 (PD-L1).

**Results:**

The patients were aged between 31 and 70 years old. The most common symptoms were abnormal vaginal bleeding and unusual vaginal discharge. HPV testing indicated that the infection rate of HPV type 18 was the highest. All patients underwent extensive hysterectomy and pelvic lymph node dissection. The progression-free survival (PFS) ranged from 3 to 112 months, with a postoperative recurrence rate of 22.7% (5/22). ISMC exhibited diverse characteristic microstructures. Immunohistochemistry results showed that the positive rates of SLC7A11 and SLC3A2 were both 91.0% (20/22). The staining intensity of SLC7A11 in frequent ISMC recurrence cases was significantly stronger than in non-recurrent ISMC cases. PD-L1 positivity was observed in 86.4% (19/22) of cases, defined as having a Combined Positive Score(CPS)≥1.

**Discussion:**

ISMC demonstrates a high rate of lymph node metastasis and a high recurrence rate, indicating strong invasiveness. Additionally, ISMC exhibits a wide morphological spectrum. SLC7A11, SLC3A2, and PD-L1 are all highly expressed in ISMC tissues. The high expression of SLC7A11 may indicate a high recurrence rate for ISMC. Immunotherapy with checkpoint inhibitors and iron death-related treatments show potential in the treatment of ISMC, with SLC7A11, SLC3A2, and PD-L1 serving as potential therapeutic targets for ISMC.

## Introduction

Invasive Stratified Mucin-producing Carcinoma (ISMC) of the cervix is a newly named type of cervical adenocarcinoma associated with Human Papilloma virus (HPV) (1, 2). Due to the limited availability of publicly documented cases in recent years, clinical data, pathological features, and molecular characteristics of ISMC are still being explored. Currently, there are no guidelines available for the treatment of ISMC. Therefore, it is essential to describe and analyze the characteristics of ISMC.

Ferroptosis was first described in 2012 as a form of programmed cell death caused by iron and lipid peroxidation-dependent reactions (3). As research on ferroptosis has advanced, it has become evident that ferroptosis plays a significant role in the inhibition of tumor progression (4–6). Ferroptosis has been demonstrated to play a significant role in the treatment of malignant tumors through radiotherapy, immunotherapy, and certain chemotherapy methods (7–10). It has been discovered that inhibitors such as ferrostatin and Liproxstatin-1 can effectively inhibit ferroptosis through distinct pathways (11). Furthermore, related drugs are currently in the process of development and testing. Ferroptosis is regulated by several key factors, including SLC7A11, which is a crucial cystine transporter and a member of the solute carrier family 7 (12). The solute carrier family 7 member 11 (SLC7A11, also known as XCT) is the primary transporter of extracellular cystine and serves as the catalytic subunit of the cystine/glutamic acid reverse transporter XC − (13–15). Currently, numerous studies have demonstrated the widespread expression of SLC7A11 in various malignant tumors. It has been found to be linked with the growth, invasion, metastasis, and poor prognosis of malignancy (16–26). Additionally, the targeted treatment strategy of SLC7A11 in cancer therapy has demonstrated its potential in the management of specific malignant tumors (12, 27). The member 2 of the Solute Carrier Family 3 (Recombinant Solute Carrier Family 3, Member 2) forms a complex with SLC7A11 to constitute the cystine/glutamate antiporter protein XC-, and SLC3A2 supports the function of SLC7A11 as a chaperone protein (13, 26). An increasing number of studies have demonstrated the significant role of SLC3A2, similar to SLC7A11, in preventing excessive lipid peroxidation within cells (8, 28). However, the expression of SLC7A11 and SLC3A2 in ISMC is currently unclear. The potential of ferroptosis-related drugs in the treatment of ISMC also requires further exploration.

Targeted therapies targeting the PD-1/PD-L1 pathway have demonstrated promising outcomes in the treatment of various types of cancer (29–32). here is multiple evidence that multiple approaches, including chemotherapy, radiation therapy, and angiogenesis inhibitors, can synergistically target PD-1/PD-L1 through various pathways, such as promoting tumor antigen release, antigen presenting cell function, or effector activity (33–39). Specifically, targeted therapies directed at the PD-1/PD-L1 pathway have demonstrated promising effects in the treatment of cervical cancer, with Pabolizumab being a notable example (40). ISMC is classified as one of the Human Papillomavirus Associated Cervical Adenocarcinoma (HPVA) in the International Endocervical Adenocarcinoma Criteria and Classification (IECC) (2). Correspondingly, the question arises as to whether anti-PD-1 plays a more significant role in HPV-positive tumors, as different clinical trials have yielded varying results (41, 42). In conclusion, further research is needed to investigate the role of targeted therapy of the PD-1/PD-L1 pathway in ISMC.

This paper provides a summary of the clinical and morphological characteristics of Invasive Stratified Mucin-producing Carcinoma (ISMC). The study analyzed data from 22 patients admitted to the Affiliated Hospital of Qingdao University between January 2013 and December 2023, including age of onset, main symptoms, HPV infection, lesion size, diagnosis method, International Federation of Obstetrics and Gynecology (FIGO) stage, concurrent cervical-related diseases, mode of operation, chemoradiation, recurrence, progression-free survival, and HE staining images of pathological tissue. In addition to previous studies on ISMC, this research further explores the expression of iron death related indicators SLC7A11 and SLC3A2. Furthermore, PD-L1 is included in this study as one potential therapeutic immune checkpoint. The purpose of this study was to summarize the clinical and morphological characteristics of ISMC and explore possible therapeutic targets.

## Methods

A total of 419 cases of cervical adenocarcinoma, pathologically diagnosed at the Affiliated Hospital of Qingdao University from January 2013 to December 2023, were included in the study. Since ISMC was recently included in the International Standard and Classification System for Cervical Adenocarcinoma (IECC), some previously diagnosed cases may need to be re-evaluated due to the short time frame of its official inclusion. After re-evaluation, 22 cases of ISMC were identified as the subjects for research.

The data of 22 patients with ISMC were collected and subjected to statistical analysis. The parameters included age of onset, main symptoms, HPV infection, lesion size, diagnostic methods, FIGO stage, complicating cervical-related disease, mode of operation, chemoradiotherapy, recurrence, progression-free survival, and HE staining images of pathological tissue.

The Formalin Fixed and Paraffin Embedded (FFPE) tissues from 22 patients with ISMC were sliced and prepared following the standard protocol. IHC staining of SLC7A11, SLC3A2, and PD-L1 was conducted, with normal cervical tissues selected for control experiments. The staining results were then evaluated.

The expression of SLC7A11 and SLC3A2 is assessed based on the intensity and extent of staining. Staining intensity is classified as 0 (no staining), 1 (mild staining), 2 (moderate staining), or 3 (strong staining), while staining extent is divided into grades based on the percentage of stained cells. The total score is calculated by multiplying the intensity score with the distribution score, with a total score greater than or equal to 4 considered positive and a total score between 0-3 considered negative. The Combined Positive Score (CPS) is used to evaluate PD-L1 expression by dividing the number of PD-L1-stained cells by the total number of viable tumor cells and multiplying by 100. At least 100 viable tumor cells are required for evaluating PD-L1 positivity. IHC staining results for SLC7A11, SLC3A2, and PD-L1 are analyzed to assess their respective expressions.

## Results

### Clinical characteristics of ISMC patients

The 22 patients ranged in age from 31 to 70 years, with an average age of 47 years. The vast majority of patients presented with symptoms of abnormal vaginal bleeding (19/22), while 3 cases exhibited abnormal fluid discharge from the vagina (3/22), and 2 cases had abnormal vaginal discharge (2/22). In addition, one patient was diagnosed with cervical adenocarcinoma through colposcopic biopsy despite exhibiting no obvious clinical symptoms. This diagnosis was prompted by a positive HPV test for type 18. Twenty patients tested positive for HPV, one tested negative, and one declined to be tested. The specific HPV subtype was identified in 11 cases. Among these, 9 cases were found to have HPV type 18 infection (9/11), 2 cases had HPV type 16 infection (2/11), and 1 case had both HPV types 18 and 45 (1/11). he majority of patients were diagnosed with ISMC through biopsy (19/22), one case was diagnosed through diagnostic conization (1/22), and one case was pathologically diagnosed as high-grade squamous intraepithelial lesion (HSIL) via biopsy, followed by diagnosis through cervical conization (1/22). Additionally, a patient who did not undergo HPV testing and cervical liquid-based cytology testing received vaginal cervical myomectomy due to vaginal bleeding. Postoperative pathology suggested ISMC (1/22). According to the latest staging of the International Federation of Gynecology and Obstetrics (FIGO), there were 15 cases in stage I (15/22), 2 cases in stage II (2/22), and 5 cases in stage IIIC1p (5/22) (**Table 1**).

**Table 1:** Clinical features of 22 patients with ISMC.

Case	Age	Symptoms	Diagnosis Methodology	FIGO Stage	HPV	Silva Pattern
1	48	Abnormal vaginal discharge	Biopsy	IIA2	18	C
2	44	Abnormal vaginal bleeding	Biopsy	IIIC1p	18	C
3	48	Abnormal vaginal bleeding	Biopsy	IB2	+	C
4	47	Abnormal vaginal bleeding	Biopsy	IB2	+	C
5	48	Abnormal vaginal bleeding	Biopsy	IB1	+	C
6	43	Abnormal vaginal bleeding	Biopsy	IB2	18、45	C
7	48	Abnormal vaginal bleeding、Abnormal vaginal discharge	Biopsy	IIA2	+	C
8	37	Abnormal vaginal discharge of fluid	Cervical conization	IIIC1p	+	C
9	52	Abnormal vaginal bleeding	Biopsy	IIIC1p	18	C
10	31	Abnormal vaginal bleeding	Biopsy	IB3	16	C
11	40	Abnormal vaginal bleeding	Biopsy	IB1	18	C
12	70	Abnormal vaginal bleeding	Biopsy	IB2	+	C
13	51	Abnormal vaginal bleeding	Biopsy	IB2	+	C
14	50	Abnormal vaginal bleeding	Biopsy	IB2	–	C
15	54	Abnormal vaginal bleeding、Abnormal vaginal discharge of fluid	Biopsy	IB1	18	C
16	48	Abnormal vaginal bleeding	Biopsy	IIIC1p	18	C
17	45	Abnormal vaginal bleeding	Biopsy	IB1	+	C
18	39	Abnormal vaginal bleeding、Abnormal vaginal discharge of fluid	Biopsy	IB2	+	C
19	49	Abnormal vaginal bleeding	Biopsy	IB2	16	C
20	56	None	Biopsy+Cervical conization	IB1	18	C
21	51	Abnormal vaginal bleeding	Biopsy	IB1	18	C
22	41	Abnormal vaginal bleeding	Cervical Myomectomy	IIIC1p	Refuse	C

### ISMC patient treatment

Among the patients (7/22), some received preoperative chemotherapy, with the majority of them undergoing 1-2 cycles of intravenous chemotherapy using a combination of paclitaxel and platinum (5/7). In addition to paclitaxel and cisplatin, a patient has added fluorouracil to the chemotherapy regimen (1/7). Another patient presented with severe vaginal bleeding and underwent paclitaxel intravenous chemotherapy and cisplatin interventional embolization therapy (1/7). All patients underwent comprehensive hysterectomy and pelvic lymph node dissection. It is worth mentioning that some patients underwent laparoscopic surgery (4/22), while others underwent open abdominal surgery (18/22). In addition, the patients underwent bilateral salpingectomy (20/22), bilateral tubal ligation and bilateral ovarian biopsy (2/22), para-aortic lymph node dissection (6/22) and presacral lymph node dissection (2/22). In terms of postoperative treatment, 13 cases underwent adjuvant chemotherapy and radiotherapy (13/22), 5 cases received only paclitaxel + platinum-based chemotherapy (5/22), 1 case underwent only radiotherapy after surgery (1/22), and 3 cases did not receive any additional treatment after surgical intervention (3/22) (**Table 2**).

**Table 2:** The ratio of inhibition of joint contamination on maize growth.

Case	Prechemotherapy	Initial chemotherapy regimen and cycle	Surgical procedure	Postoperative treatment
1	+	Paclitaxel + cisplatin,Intravenous chemotherapy,2 cycle	Open radical hysterectomy+Bilateral adnexectomy+Pelvic lymph node dissection+腹主动脉旁淋巴结	Paclitaxel + Platinum,chemotherapy
2	+	Docetaxel+carboplatin,Intravenous chemotherapy,1 cycle	Laparoscopic radical hysterectomy+Bilateral adnexectomy+Pelvic lymph node dissection	Docetaxel+carboplatin,chemotherapy、radiotherapy
3	–	–	Open radical hysterectomy+Bilateral adnexectomy+Pelvic lymph node dissection+ retroperitoneal lymph node dissection of the abdominal aorta+Sentinel lymph node tracing	Albumin paclitaxel+carboplatin,chemotherapy
4	+	Paclitaxel liposomes+nedaplatin,1 cycle Docetaxel,Intravenous chemotherapy+cisplatin,Interventional embolization,1 cycle	Open radical hysterectomy+Bilateral adnexectomy+Pelvic lymph node dissection	Docetaxel+carboplatin、radiotherapy
5	+	Paclitaxel + cisplatin+fluorouracil,Intravenous chemotherapy,1 cycle	Open radical hysterectomy+Bilateral adnexectomy+Pelvic lymph node dissection	None
6	–	–	Laparoscopic radical hysterectomy+Bilateral adnexectomy+Pelvic lymph node dissection+ retroperitoneal lymph node dissection of the abdominal aorta+ presacral lymph node dissection	Paclitaxel + Platinum,chemotherapy
7	+	Paclitaxel liposomes+carboplatin,Intravenous chemotherapy,2 cycle	Open radical hysterectomy+Bilateral adnexectomy+Pelvic lymph node dissection	Paclitaxel + Platinum,chemotherapy
8	–	–	Open radical hysterectomy+Bilateral salpingectomy+Pelvic lymph node dissection+ retroperitoneal lymph node dissection of the abdominal aorta+bilateral ovarian biopsy procedure	Paclitaxel + Platinum,chemotherapy、radiotherapy
9	–	–	Open radical hysterectomy+Bilateral adnexectomy+Pelvic lymph node dissection	Paclitaxel + Platinum,chemotherapy、radiotherapy
10	+	Paclitaxel + cisplatin,Intravenous chemotherapy,1 cycle	Laparoscopic radical hysterectomy+Bilateral adnexectomy+Pelvic lymph node dissection+ retroperitoneal lymph node dissection of the abdominal aorta	Paclitaxel + Platinum,chemotherapy、radiotherapy
11	–	–	Open radical hysterectomy+Bilateral adnexectomy+Pelvic lymph node dissection	None
12	–	–	Open radical hysterectomy+Bilateral adnexectomy+Pelvic lymph node dissection	Paclitaxel + Platinum,chemotherapy、radiotherapy
13	–	–	Open radical hysterectomy+Bilateral adnexectomy+Pelvic lymph node dissection	Docetaxel+cisplatin,chemotherapy、radiotherapy
14	–	–	Open radical hysterectomy+Bilateral adnexectomy+Pelvic lymph node dissection	None
15	–	–	Open radical hysterectomy+Bilateral adnexectomy+Pelvic lymph node dissection	Albumin paclitaxel+carboplatin,chemotherapy,3 cycle Docetaxel+carboplatin,1 cycle、radiotherapy
16	+	Paclitaxel+carboplatin,2 cycle	Open radical hysterectomy+Bilateral adnexectomy+Pelvic lymph node dissection	Paclitaxel + Platinum,chemotherapy、radiotherapy
17	–	–	Laparoscopic radical hysterectomy+Bilateral adnexectomy+Pelvic lymph node dissection	Paclitaxel + Platinum,chemotherapy
18	–	–	Open radical hysterectomy+Bilateral salpingectomy+Pelvic lymph node dissection+ retroperitoneal lymph node dissection of the abdominal aorta+bilateral ovarian biopsy procedure	Paclitaxel + Platinum,chemotherapy、radiotherapy
19	–	–	Open radical hysterectomy+Bilateral adnexectomy+Pelvic lymph node dissection	radiotherapy
20	–	–	Open radical hysterectomy+Bilateral adnexectomy+Pelvic lymph node dissection	Paclitaxel + Platinum,chemotherapy、radiotherapy
21	–	–	Open radical hysterectomy+Bilateral adnexectomy+Pelvic lymph node dissection	Paclitaxel + Platinum,chemotherapy、radiotherapy
22	–	–	Open radical hysterectomy+Bilateral adnexectomy+Pelvic lymph node dissection+ presacral lymph node dissection	Paclitaxel + Platinum,chemotherapy、radiotherapy

### Follow-up of ISMC patients

In this study, the progression-free survival (PFS) of all ISMC patients ranged from 3 to 112 months (average 33.95 months), with a 1-year recurrence rate of 4.5% and a 2-year recurrence rate reaching 18.2%. This study included a total of 5 cases of postoperative recurrence. Among them, 2 cases showed elevated tumor markers without any imaging evidence of disease progression, and both patients continued regular follow-up. One patient had pelvic lesions identified on PET-CT and chose to undergo oral traditional Chinese medicine treatment. Additionally, 2 cases of malignant tumors invaded the urinary system and resulted in hydronephrosis; one case experienced three recurrences, with the first two recurrences treated with paclitaxel, cisplatin, and fluorouracil chemotherapy via intravenous infusion. The third recurrence led to hemorrhagic shock caused by vaginal bleeding, requiring a sigmoid colostomy due to rectovaginal fistula. The other case underwent transurethral resection of bladder lesions and percutaneous nephrostomy for drainage before receiving traditional Chinese medicine treatment postoperatively (**Table 3**).

**Table 3:** Follow-up information of 22 patients with ISMC.

Case	recrudescence	PFS(month)	Recurrent situation	Post-relapse treatment
1	–	36		–
2	+	9	2018.10 CT results: Presence of a lesion in the surgical site with surrounding invasion; observed dilatation of the left ureter; thickening of the bladder wall, potentially suggestive of tumor invasion; Presence of a nodule near the sigmoid colon, indicating a high probability of metastatic tumor. 2019.11 CEA:168.30;SCC:6.79ng/mL;CA125:4141.0U/mL 2020.07 Vaginal bleeding, hemorrhagic shock, and rectovaginal fistula.	2018.10 Placement of a ureteral stent; administration of four cycles of docetaxel plus cisplatin, and three cycles of 5-fluorouracil plus cisplatin. 2019.11 Three rounds of chemotherapy utilizing albumin-bound paclitaxel. 2020.07 Anti-shock, single-cavity colostomy surgery for the sigmoid colon.
3	–	30		–
4	+	17	2016.11 CA125:125.6U/mL;SCC 3.00ng/mL PET-CT scan reveals a small nodular density measuring 6mm in diameter in the left pelvic soft tissue.	Treatment with traditional Chinese medicine
5	–	91		–
6	–	43		–
7	–	112		–
8	–	9		–
9	–	10		–
10	–	64		–
11	–	95		–
12	–	19		–
13	–	8		–
14	–	13		–
15	–	10		–
16	+	29	CEA:62.1ng/mL The CT scan of the pelvis reveals normal findings.	unknown
17	–	62		–
18	–	39		–
19	+	24	2021.01 CEA3.77ng/mL 2021.08 CEA5.06ng/mL 2021.09 CEA4.77ng/mL 2022.08 CEA8.38ng/mL	–
20	–	10		–
21	+	14	Frequent urination and left kidney pain, with a pathological diagnosis of poorly differentiated carcinoma (bladder tumor). Considering the morphology and clinical history, there is suspicion of cervical infiltrating multilayer mucin secretion in the cancer. Please consider in conjunction with clinical findings.	Transurethral resection of bladder tumor and percutaneous nephrostomy for drainage, followed by postoperative treatment with traditional Chinese medicine.
22	–	3		–

### Macroscopic characteristics of ISMC

Overall morphological features indicate that ISMC presents as ulcerative, cauliflower-like, or polypoid tumors with rough cut surface, and grayish-white or grayish-yellow coloration. The maximum diameter of the tumor is 7-60mm, with an average of 28mm (**Table 4**).

**Table 4:** General characteristics of ISMC in 22 cases.

Case	Macro performance	Tumor size(cm)
1	Ulcerative mass	4.00
2	Ulcerative mass	3.00
3	Polypoid mass	3.00
4	Polypoid mass	3.00
5	The cauliflower-shaped protuberance	1.50
6	Ulcerative mass	2.00
7	Ulcerative mass	6.00
8	Polypoid mass	3.00
9	Ulcerative mass	3.00
10	Ulcerative mass	3.00
11	Ulcerative mass	1.80
12	Polypoid mass	3.60
13	Ulcerative mass	2.50
14	Ulcerative mass	3.00
15	Ulcerative mass	1.90
16	The cauliflower-shaped protuberance	4.00
17	Polypoid mass	1.10
18	The cauliflower-shaped protuberance	2.50
19	The cauliflower-shaped protuberance	3.00
20	Polypoid mass	0.70
21	Polypoid mass	1.00
22	Ulcerative mass	5.00

### Microstructural characteristics of ISMC

**Figure 1:**
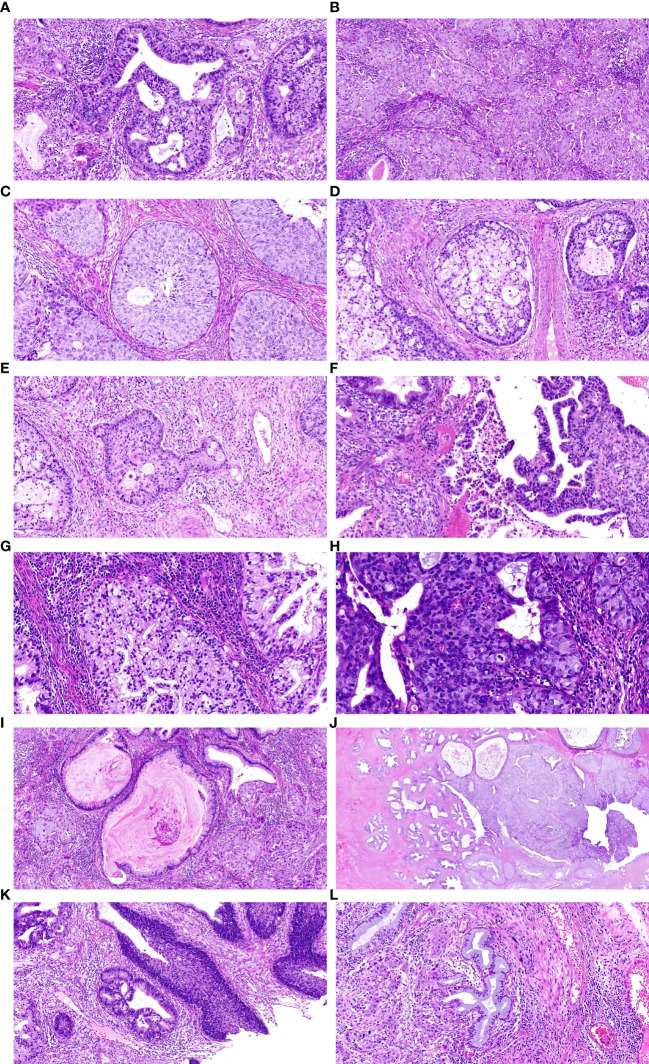
**(A)** shows a typical SMILE lesion, **(B)** displays a “rich in mucin” SMILE with abundant mucins in the cytoplasm. **(C)** illustrates a “mucin-poor” SMILE with relatively low levels of mucins present in the cytoplasm, **(D)** presents a typical ISMC lesion. **(E)** demonstrates infiltrative mucinous carcinoma cells nests exhibiting glandular-like changes within surrounding cell nuclei, **(F)** showcases abundant presence of mucins within the cytoplasm along with infiltration by peripheral neutrophils. **(G)** presents stratified epithelium displaying nuclear atypia and intracytoplasmic vacuoles filled with mucus, **(H)** represents apoptotic bodies, **(I)**indicates an extracellular pool of mucus, **(J)** shows SMILE involving endocervical glands on the left transitioning into an ISMC lesion on the right, **(K)** displays combination of ISMC and HSIL **(L)** revealing concurrent development of original site adenocarcinoma within an ISMC case.

In the HE stained sections of ISMC cases, a stratified mucin-producing intraepithelial lesion (SMILE) involving the cervical glands can be observed (**Figure 1A**). Unlike traditional squamous cell carcinoma and adenocarcinoma, this type presents as a mixture of stratified epithelial cells and immature nuclei, similar to high-grade squamous intraepithelial lesion (HSIL), but also shows varying proportions of intracytoplasmic vacuoles containing mucin. The quantity of intracellular adhesive proteins ranges from “rich in adhesive proteins” lesions, which show a large amount of adhesive proteins in most cells, to “deficient in adhesive proteins” lesions, which only display a relatively limited number of cells containing adhesive proteins (**Figures 1B, C**). Under low magnification, it is clearly observed that the cells producing adhesive proteins are distributed throughout the entire epithelial cells. The presence of invasive nests formed by multi-layered mucous cells with peripheral fence-like patterns is considered as clear evidence of invasion (**Figures 1D, E**). Neutrophil infiltration can be observed around the cancer nests (**Figure 1F**). Peripheral to some lesions, there is evidence of nuclear atypia in the stratified epithelium. Within the lesion, mucin vacuoles can be observed within the cytoplasm of cancer cells (**Figure 1G**). In the lesion, membrane shrinkage and internal invagination can be found, dividing and enclosing the cytoplasm to form vesicular bodies, known as apoptotic bodies (**Figure 1H**). Some lesions present extravasated mucin pools (**Figure 1I**). The invasion of cervical intraepithelial neoplasia by SMLIE can be observed in multiple slices, adjacent to the ISMC lesion (**Figure 1J**). This indirectly proves their progressive relationship. In this study, out of the 22 cases of ISMC, 2 cases were found to be combined with HISL (**Figure 1K**), and 3 cases were found to be combined with Adenocarcinoma *In Situ* (AIS) (**Figure 1L**). These findings were all demonstrated in HE sections.

### Immunohistochemical staining results of SLC7A11 and SLC3A2

**Figure 2:**
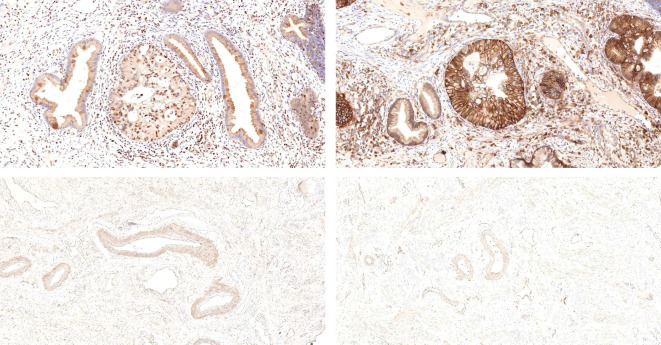
ISMC tissue staining for SLC7A11 and SLC3A2 is depicted in the upper left and upper right, while staining of SLC7A11 and SLC3A2 in normal cervical tissue is shown in the lower left and lower right.

The expression of SLC7A11 demonstrated a high positivity rate, reaching 91.0% (20/22) (**Table 5**). Its expression was observed in the cytoplasm and cell membrane, with no apparent staining in the cell nucleus (**Figure 2**). Similarly, the expression rate of SLC3A2 also reached 91.0% (20/22) (**Table 5**), and its expression was localized to the cell membrane, with less prominent staining in the cell nucleus and cytoplasm (**Figure 2**).

**Table 5:** Immunohistochemical staining results of SLC7A11 and SLC3A2 in 22 patients with ISMC.

Case	SLC7A11			SLC3A2		
	Staining intensity	Percentage of stained cells	Evaluation	Staining intensity	Percentage of stained cells	Evaluation
1	2	3	6	3	3	9
2	3	3	9	3	3	9
3	3	3	9	3	3	9
4	3	3	9	3	3	9
5	3	3	9	3	3	9
6	3	3	9	3	3	9
7	3	3	9	3	3	9
8	2	3	6	3	3	9
9	2	3	6	3	3	9
10	2	3	6	3	3	9
11	1	3	3	3	3	9
12	3	3	9	3	3	9
13	1	3	3	3	3	9
14	3	3	9	3	2	9
15	2	3	6	2	3	6
16	3	3	9	3	3	9
17	3	3	9	3	1	3
18	3	3	9	3	3	9
19	3	3	9	1	3	3
20	3	3	9	3	3	9
21	3	3	9	3	3	9
22	3	3	9	3	3	9

### Comparison of immunohistochemical staining results for SLC7A11 and SLC3A2 in frequent recurrent ISMC cases versus non-recurrent ISMC cases

**Figure 3:**
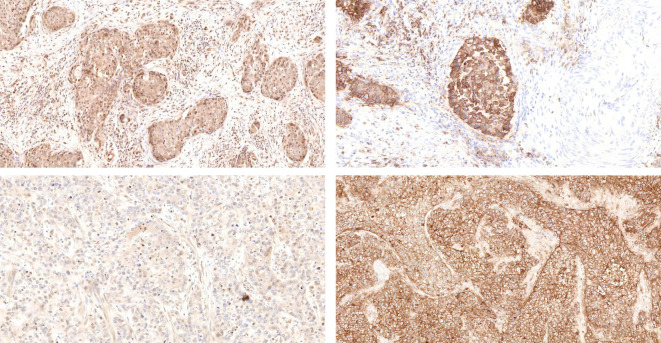
Comparison of tissue staining for SLC7A11 (top left) and SLC3A2 (top right) in cases of frequent recurrent ISMC is contrasted with tissue staining for SLC7A11 (bottom left) and SLC3A2 (bottom right) in non-recurrent ISMC cases.

Ever since Bannai and Kitamura first identified it in 1980, there has been a significant rise in the number of studies showing the extensive presence of SLC7A11 in different types of cancer, along with its various impacts on cancer development, spread, migration, and poor prognosis (16–26, 43–47). In this study, a comparison of the staining for SLC7A11 and SLC3A2 was conducted between ISMC cases with three recurrences and those without recurrence. The staining intensity of SLC7A11 in frequent ISMC recurrence cases was significantly stronger than that in non-recurrent ISMC cases, while the difference in staining intensity of SLC3A2 between the two was not significant (**Figure 3**).

### Immunohistochemical staining results for PD-L1

**Figure 4:**
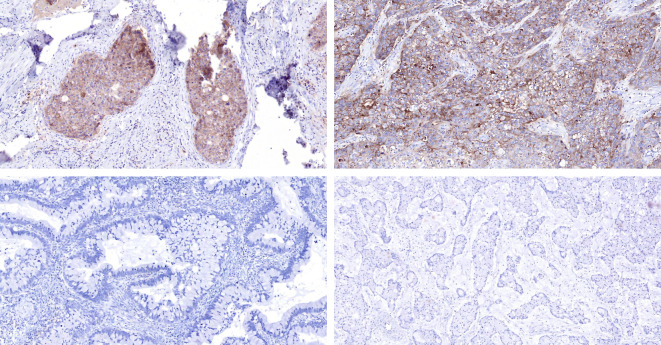
Comparison is made between tissue staining of PD-L1 CPS scores of 100 (top left) and 98 (top right) in ISMC cases, and tissue staining of PD-L1 CPS scores <1 (bottom left, bottom right) in ISMC cases.

The immunohistochemistry results revealed that the positive rate of PD-L1 (CPS≥1) was 86.4% (19/22), with a maximum CPS score of 100 (**Table 6**; **Figure 4**). PD-L1 was found to be located in both tumor cells and tumor-associated immune cells within the tumor stroma.

**Table 6:** Immunohistochemical staining of PD-L1 in 22 patients with ISMC.

Case	PD-L1
	CPS score
1	5
2	100
3	5
4	30
5	<1
6	20
7	70
8	98
9	5
10	<1
11	3
12	11
13	25
14	20
15	10
16	90
17	10
18	5
19	10
20	<1
21	3
22	10

## Discussion

The ISMC has been recently discovered as a type of cervical adenocarcinoma associated with HPV. Prior to ISMC being officially included in IECC, these instances were often incorrectly diagnosed as adenocarcinoma, squamous cell carcinoma, or different forms of cervical intraepithelial neoplasia (48). At present, there is limited comprehensive statistical information available regarding the prevalence of IECC. An examination of 200 instances of cervical squamous cell carcinoma and adenocarcinoma found that ISMC represents 5% of cases (49). During the same time period, our hospital identified a total of 419 cases of cervical adenocarcinoma, with ISMC accounting for 5.3% (22/419) of the cases. Previous studies have shown that ISMC displays more aggressive clinical and pathological features when compared to conventional HPV-related cervical adenocarcinoma (50–52). The study found that 22.7% (5/22) of ISMC cases had lymph node metastasis, demonstrating its significant invasiveness and emphasizing the importance of precise diagnosis for ISMC.

As a result of the small number of documented cases and lack of clear understanding regarding its natural progression, there is currently no widely accepted standard for treating and predicting the outcome of ISMC. Additionally, there is limited available evidence to use as a reference. The clinical staging, rather than the histological subtype, dictates the prognosis and treatment for patients with cervical cancer. The survival rates differ greatly depending on the stage of the disease. Patients with cervical adenocarcinoma show varying survival rates at different stages, including a 5-year survival rate of 89.8% for stage I, 79.1% for stage II, 47.7% for stage III, and 42.4% for stage IV (53). The majority of research has indicated that tumor size, tumor grade, and lymph node metastasis are all considered as separate prognostic factors. Furthermore, there is also evidence to suggest that marital status may be an independent prognostic factor (53). In the initial phases of the illness (IA1 to IB1), local surgical techniques such as conization, radical hysterectomy, and evaluation of lymph node status are frequently employed as primary treatment approaches (53). Radiotherapy is a potential option for patients who are not eligible for surgery or refuse surgical treatment. The typical course of treatment for individuals with stage IB2-IVA cervical cancer includes simultaneous cisplatin-based chemoradiotherapy in conjunction with brachytherapy (54). The therapy is suitable for individuals with glandular and squamous conditions, and additional research using clinical samples is needed to assess its effectiveness in preventing the spread of cancer triggered by aggressive laminin.

The age at which ISMC patients in this study first experienced symptoms varied from 31 to 70 years old. A different study on ISMC found that the average age of onset ranged from 37 to 75 years old, with a mean age of 50.5 years, suggesting similar findings in terms of the distribution of ages (49). This indicates that there is a potential for ISMC to occur in young women as well. The prevailing agreement is that ISMC is strongly linked to high-risk HPV, specifically types 18 and 16 (49, 55). This research supports the above perspective by showing that 10 out of 11 cases were infected with HPV 18, and only 2 out of 11 cases were infected with HPV 16, when specific HPV genotypes could be identified. HPV is present in most of the cases that are publicly accessible (2, 50, 55). Not all instances of ISMC can be identified through HPV infection. In this research, one out of 21 cases showed a negative result for HPV, and similar occurrences of undetected HPV have also been documented in other studies (3 out of 12) (56). The IECC defines ISMC as cervical cancer related to HPV, and current evidence supports this idea. However, more research is needed to understand the underlying mechanisms. It is also important to investigate why some cases of HPV go undetected, as the limited sensitivity of HPV detection alone may not fully explain this phenomenon. The prognostic significance of different high-risk HPV genotypes, particularly HPV16, 18, and 45, in relation to cervical cancer remains uncertain. Some research has indicated positive outcomes for cervical cancer associated with HPV16 and/or 18 positivity (57–61), while others have suggested a less favorable prognosis (62–72). Furthermore, certain studies have found that high-risk HPV genotypes do not hold prognostic value (73–76). The notable distinctions in clinical characteristics between cervical squamous cell carcinoma and cervical adenocarcinoma, as well as HPV-related and non-HPV-related endocervical adenocarcinomas, underscore the significance of acknowledging inherent prejudices in research investigations. Given the substantial differences in clinical behavior between these histological types and etiologies of cervical cancer, it may be optimal to investigate the prognostic significance of high-risk HPV genotypes based on these factors. Due to the infrequent occurrence of ISMC cases, there is a restricted amount of documented cases accessible. Our objective is to gather a sufficient quantity of ISMC cases for the purpose of analyzing the predictive importance of high-risk HPV genotypes.

It has been documented that PD-L1 expression is observed in tumor cells and different types of immune cells in both cervical squamous cell carcinoma and adenocarcinoma (77). Compared to cervical endometrial adenocarcinoma, squamous cell carcinoma exhibits a higher rate of PD-L1 positivity and an increased presence of immune cells expressing PD-L1. Furthermore, studies suggest that patients with tumor-associated macrophages showing PD-L1 expression in cervical endometrial adenocarcinoma experience a significantly shorter period of progression-free survival compared to those with tumors lacking PD-L1 expression (77). Recent studies of 27 instances of endometrial adenocarcinoma samples found that positive PD-L1 expression was observed in tumor cells in 3.7% of cases, while immune cells showed positive PD-L1 expression in 7.4% of cases (78). The research on cervical adenocarcinoma found no link between the presence of high-risk HPV genotypes and the levels of PD-L1 expression (79). While the occurrence of PD-L1 expression is infrequent, its detection in either tumor cells or immune cells is strongly associated with unfavorable outcomes for individuals diagnosed with cervical adenocarcinoma (78). The presence of PD-L1 expression could indicate the tumor’s defensive reaction to immune attack by compensatory upregulation of PD-L1 or adaptive immune resistance (80, 81). Overall clinical outcomes often depend on the balance between tumor-induced immune responses and immunosuppressive mechanisms, such as the interaction of programmed cell death protein 1/PD-L1. The simultaneous increase in CD8+ tumor-infiltrating lymphocytes and PDL1 expression in cervical adenocarcinoma suggests a tendency towards immune resistance (82), leading to more aggressive tumor behavior. In this research, PD-L1 expression in ISMC patients was evaluated using the CPS system. The results of immunohistochemistry showed that 86.4% (19/22) of patients had a positive rate of PD-L1 (CPS≥1), with the highest CPS score being 98. PD-L1 was detected in both tumor cells and tumor-associated immune cells within the tumor stroma. This finding suggests that high levels of PD-L1 expression may be associated with aggressive behavior and a worse prognosis in ISMC. The trial approved by the FDA in the United States has shown that pembrolizumab, an anti-PD-1 inhibitor, is effective in treating advanced cervical cancer patients with PD-L1 positive status (CPISMC 1) and leads to improved outcomes for patients with recurrent or metastatic cervical cancer (83). These results indicate that PD-1/PD-L1 immunotherapy may be beneficial for these patients.

SLC7A11 serves as the catalytic component of the amino acid transportation system XC-, which is accountable for taking in extracellular cystine molecules and converting them into cysteine, ultimately producing glutathione (84). GSH acts as a strong remover of lipid peroxidation and is an essential coenzyme for the activity of glutathione peroxidase 4 (Gpx4). It has a crucial function in eliminating phospholipid peroxides and protecting cells from ferroptosis (85). As a result, the SLC7A11-GSH system acts as the primary defense mechanism against ferroptosis and plays a crucial role in regulating cellular iron balance (15). Inhibiting SLC7A11 may hinder the production of cysteine and the synthesis of glutathione, potentially resulting in cell death caused by excessive accumulation of lipid peroxidation due to high levels of iron (86). Cysteine can be partially replenished through the sulfurization pathway or alternative non-selective amino acid transporters (87). Cancer cells require a large amount of cysteine and glutathione to neutralize the high levels of reactive oxygen species in their cells, resulting in an upregulation of SLC7A11 as a component of their reliance on nutrients (12, 88). SLC7A11 has been found to be overexpressed in many types of human cancers, and specifically inhibiting SLC7A11 shows a high level of responsiveness (89). The inhibition of the SLC7A11-GSH pathway has been shown to have a significant anti-tumor effect in various types of human cancers (90). Our study indicates that gaining a deeper understanding of the functions of cysteine and SLC7A11 in controlling the redox activity proteins and their interactions could present a promising strategy for treating cancer. The high rate of SLC7A11 expression in ISMC, which aligns with its significant invasiveness, metastasis, and unfavorable prognosis, offers new perspectives on the currently uncertain treatment of ISMC. It is worth noting that there are indications that the overexpression of SLC7A11 in cancerous tumors can increase the activity of the PD-L1 pathway, resulting in the infiltration of tumor-associated macrophages (TAM) and myeloid-derived suppressor cells (MDSC) within the tumor. Inhibiting this oncogenic pathway could potentially serve as an effective therapeutic approach for preventing SLC7A11-mediated metastasis of malignant tumors (91).

SLC3A2, also referred to as CD98, CD98hc or 4F2hc, is a type II transmembrane protein with a molecular weight of 80 kDa that is mainly positioned facing the outside of the cell (92). SLC3A2 is essential for regulating ferroptosis, apoptosis, and proliferation in cancer cells by interacting with XCT (90, 93, 94). The increased expression of SLC3A2 has been associated with the initiation and advancement of various forms of cancer because of its wide range of molecular activities. Previous studies have indicated that SLC3A2 plays a role in the progression of oral cancer by promoting cell growth and suppressing programmed cell death in individuals (94). Our research aims to fill the gap in literature regarding the expression of SLC3A2 in ISMC, and also illustrates the role of the XCT system, consisting of SLC7A11 and SLC3A2, in the iron death process. These findings suggest that iron death-related therapy may be a potential treatment for ISMC, indicating that this mechanism is present in these cells.

Our research has certain limitations, as a considerable number of ISMC cases had already undergone surgery before the formal introduction of the ISMC concept, which prevented us from obtaining the latest tissues and cells of ISMC. Therefore, we were unable to conduct further *in vivo* and *in vitro* experiments. Due to the rarity of ISMC, our study had a limited number of cases, which restricted us from conducting some statistical studies, such as investigating whether HPV18 infection is associated with the expression of SLC7A11, SLC3A2 and PD-L1. If given the opportunity, we will make efforts to increase the number of ISMC cases and collect new ISMC tissues for improvement.

In summary, this research has analyzed clinical data and pathological characteristics of cervical endometrioid adenocarcinoma. Due to the varied appearance of this cancer type, a thorough examination is crucial for an accurate diagnosis, especially for less experienced pathologists. Even seasoned pathologists may face difficulties in distinguishing cervical endometrioid adenocarcinoma from other types. The frequent occurrence of lymph node and distant metastasis, as well as postoperative recurrence, indicates the aggressive nature and poor prognosis associated with cervical endometrioid adenocarcinoma. Based on the findings of the immunohistochemistry study, it is possible that ISMC patients could benefit from immune checkpoint inhibitors that target PD-L1/PD-1 and ferroptosis-related therapy aimed at SLC7A11.

## Data Availability

The original contributions presented in the study are included in the article/supplementary material. Further inquiries can be directed to the corresponding authors.
